# Genetic variations in the *Myostatin* gene affecting growth traits in sheep

**DOI:** 10.14202/vetworld.2021.475-482

**Published:** 2021-02-23

**Authors:** Noha M. Osman, Heba I. Shafey, Mohamed A. Abdelhafez, Ahmed M. Sallam, Karima F. Mahrous

**Affiliations:** 1 Department of Cell Biology, National Research Centre, El Buhouth Street, 12311, Dokki, Egypt; 2 Animal and Poultry Production Division, Desert Research Center, 11753, Mataryia, Egypt

**Keywords:** growth traits, *Myostatin* gene, polymorphism, sheep

## Abstract

**Background and Aim:**

Sheep productivity in developing countries is crucial, as this animal is an essential source of meat and wool. *Myostatin* (*MSTN*) plays an important role in the regulation of muscle mass through the regulation of muscle growth, differentiation, and regeneration. The present study sought to investigate genetic variation in the first intron of the *MSTN* gene and the association of variants with growth traits in major sheep breeds in Egypt (Barki, Ossimi, and Rahmani) and Saudi Arabia (Najdi) using polymerase chain reaction (PCR) and sequencing.

**Materials and Methods:**

Blood samples were collected, and DNA was extracted from 75 animals. A 386 bp fragment in the first intron of the *MSTN* gene was amplified using PCR. Polymorphic sites were detected using direct sequencing and then correlated with growth traits using a general linear model.

**Results:**

Sequence analysis of the first intron of *MSTN* gene identified six single-nucleotide polymorphisms (SNPs) in the studied breeds. Four mutual SNPs were determined: c.18 G>T, c.241 T>C, c.243 G>A, and c.259 G>T. In addition, two SNPs c.159 A>T and c.173 T>G were monomorphic (AA and TT, respectively) in the Ossimi, Rahmani, and Najdi breeds and polymorphic in the Barki breed. The association analysis revealed that the c.18 G>T and c.241 C>T significantly associated (p<0.05) with birth weight and average daily weight gain, respectively.

**Conclusion:**

Our results strongly support *MSTN* as a candidate gene for marker-assisted selection in sheep breeding programs. Furthermore, the identified variants may be considered as putative markers to improve growth traits in sheep.

## Introduction

Sheep are an essential component of the agricultural sector in Egypt, as well as worldwide. Conventionally, sheep supply small and marginal breeders with meat, milk, and wool products. In Egypt, sheep are an important source of meat production, contributing approximately 6% of total red meat produced [1]. Based on the total number of sheep, Barki, Ossimi, and Rahmani are the major sheep breeds in Egypt distributed along the western Mediterranean coastal region, the middle of Egypt, and the Northern Nile delta [2]. Substantial variations distinguish between these breeds in phenotypic and productive characteristics [3]. Conversely, Najdi sheep are the prime local breed in the eastern province of Saudi Arabia, and it has the most favorable meat with the most desired taste among all breeds in Saudi Arabia [4].

The performance traits of animals (e.g., growth performance) have a direct impact on the profitability of any animal production enterprise; therefore, these traits have been targeted by several sheep breeding programs in different countries [5]. The genetic basis of any such performance trait should be understood. Growth performance as a quantitative trait is controlled by many genes, one of which is *Myostatin* (*MSTN*) [6].

*MSTN*, also known as growth and differentiation factor 8, is a member of the transforming growth factor-β superfamily and acts as a negative regulator of skeletal muscle growth [7]. It is located at the end of the long arm of chromosome 2 (2q32.2) in sheep (*Ovis aries*) and comprises three exons and two introns [8]. It has previously been recommended as a candidate gene to improve muscle production in sheep [9]. Moreover, the association of *MTSN* polymorphisms with several muscle-related traits has been reported in other livestock, such as cattle [10], chickens [11], horses [12], and rabbits [13]. Importantly, the “double-muscling phenomenon” observed in different species is a result of mutations in *MSTN* that disrupts its expression, resulting in a completely non-functional protein. This has great potential to enhance muscle growth, leading to dramatic muscularity [14-16].

Association analysis using single-nucleotide polymorphisms (SNPs) is the most effective approach to identify genetic markers potentially related to a trait of interest [17]. This involves screening candidate genes, which may be biologically related to the desired trait, for putative mutations, and consequently correlating these results with accurate phenotypes of a group of individuals [18,19]. The identified genetic markers may be useful in selection and breeding programs in livestock [20]. Variations in the non-coding regions of the *MSTN* gene have been found to relate to muscle growth and meat quality traits in sheep [21,22], which can be attributed to their effects on the regulatory elements of the gene itself [23].

Several studies have reported that the variations in *MSTN* have been associated with increased skeletal muscle mass in sheep [9,24] and muscular yield commercially [25,26]. Therefore, *MSTN* in farm animals should be considered to identify the appropriate animals for selection programs, especially marker-assisted selection for economic traits [27]. In this study, a combination of polymerase chain reaction (PCR) and DNA sequencing was used to ascertain genetic variation in intron 1 of the *MSTN* gene in different sheep breeds and their association with growth traits such as birth weight (BW), final weight (FW), and average daily weight gain (ADG).

## Materials and Methods

### Ethical approval

This study does not require ethical approval; however, samples were collected as per standard sample collection procedure without any harm to animals. The authors obtained consent from sheep farm owners for sample collection.

### Study period and location

The samples were collected from August to November 2018 from two animal production farms that belong to the Faculty of Agriculture, Ain Shams University, and the Faculty of Agriculture, Al-Azhar University, Egypt. DNA isolation, PCR and Sequence analysis were carried out from December 2018 to August 2019.

### Animals and blood sampling

The present study was conducted on 75 animals, including 60 animals from three different Egyptian sheep breeds and 15 animals from a Saudi Arabian Najdi breed. The Egyptian sheep comprised 40 females and 20 males from Barki (17), Rahmani (21), and Ossimi (22) breeds. Barki sheep were maintained at Nubaria Farm, National Research Centre, Egypt. Rahmani and Ossimi sheep were sourced from two animal production farms belonging to the Faculty of Agriculture, Ain Shams University, and the Faculty of Agriculture, Al-Azhar University, Egypt. Phenotypic data including BW, FW at slaughter, and ADG were recorded for each animal of the Egyptian breeds. Finally, blood samples were collected from the jugular veins of all 75 animals in vacuum tubes containing 0.25% of the anticoagulant ethylenediaminetetraacetic acid. These samples were stored at −80°C until DNA extraction.

### DNA extraction

Using the salting out procedure described by Miller *et al*. [28], genomic DNA was extracted from whole blood. The DNA concentrations were determined using a NanoDrop 1000 (Thermo Scientific) and then were adjusted to concentrations of 50 ng/μL for PCR.

### PCR amplification

Based on the primers published by Sjakste *et al*. [23], the following forward and reverse primers were used for PCR amplification of the first intron of the *MSTN* gene: Forward 5′-GAAACGGTCATTACCATGC-3′ and reverse 5′-CATTTGGTTGCCTGAAATATG-3′. The 25 μL PCR reaction mixture consisted of 3 μL (150 ng) template DNA, 1 μL forward primer (10 μM), 1 μL reverse primer (10 μM), 12.5 μL 2× PCR master mix, and 7.5 μL nuclease-free water. The reaction was cycled at the following conditions: Initial denaturation for 5 min at 94°C, followed by 35 cycles of denaturation at 94°C for 1 min, annealing at 62°C for 1 min, and extension at 72°C for 1 min, and a final extension for 5 min at 72°C. The PCR product was analyzed by electrophoresis on 2% agarose gel.

### Sequence analysis and SNPs identification

Purified PCR products were sequenced by Macrogen, Incorporated (South Korea) using forward and reverse primers. The specificity of the nucleotide sequences was determined using Basic Local Alignment Search Tool (BLAST, https://blast.ncbi.nlm.nih.gov/Blast.cgi) [29]. Sequences were analyzed through multiple alignments using Clustal Omega (https://www.ebi.ac.uk/Tools/msa/clustalo/) [30] to determine polymorphic sites, which were confirmed through the visual examination of sequence charts.

### Statistical analysis

The association between identified *MSTN* genotypes and the studied traits was determined using the general linear model process in SAS (SAS Version 8.2, SAS Institute, Cary, NC). The following model was used to assess the significance of associations:

Y_ijk_ = μ+B_i_+G_j_+H_k_+e_ijk_,

Where, Y_ijk_=the trait of interest (BW, FW, and ADG); μ=the overall mean; B_i_=the fixed effect of the breed (3 levels); G_j_=the fixed effect of the *i*^th^ genotype corresponds to each SNP independently; H_k_=the fixed effect of the *k*^th^ sex of animal (2 levels); and e_ijk_=random error. The random error was assumed to be normally distributed with a mean equal zero and variance equals δ^2^_e_.

## Results

### Descriptive statistics

Table-1 presents the estimates of least square means±standard deviations and minimum and maximum BW, FW, and ADG for the studied breeds. In general, BW ranged from 2.5 to 4.1 kg; FW, from 37 to 63 kg; and ADG, from 68 to 142 g/day. A higher average BW was observed in Rahmani sheep (3.4 kg) and Ossimi sheep (3.46 kg) than in Barki sheep (2.93 kg). Similarly, the FW was higher in Rahmani sheep (51.58 kg) and Ossimi sheep (48.11 kg) than in Barki sheep (43.62 kg). Similarly, a higher ADG was observed in Rahmani sheep (98.83 g/day) and Ossimi sheep (100.66 g/day) than in Barki sheep (90.12 g/day).

**Table-1:** Descriptive statistics of the studied traits.

Trait^1^	Average	SD^2^	Minimum	Maximum
Barki				
BW (kg)	2.93	0.29	2.5	3.2
FW (kg)	43.62	6.1	37	53
ADG (g/day)	90.125	11.97	68	104
Rahmani				
BW (kg)	3.4	0.54	2.8	4.1
FW (kg)	51.58	8.3	43	63
ADG (g/day)	98.83	28.62	75	142
Ossimi				
BW (kg)	3.46	0.28	3	3.8
FW (kg)	48.11	5.76	41	56
ADG (g/day)	100.66	23.76	78	131

1 BW=Birth weight (kg), FW=Full weight (kg), ADG=Average daily gain (g/day). SD

2 =Standard deviation

### Effect of breed and sex

Analyses of variance indicated that neither the fixed effect of the breed nor the sex of the animal had significant effects (p<0.05) on the BW. The breed tended to have a significant effect (p=0.07) on FW, whereas the sex of the animal had a highly significant effect on the FW (p=0.001). Similarly, the breed did not affect ADG, although sex had a significant effect (p=0.002) on ADG, as male lambs had a significantly higher ADG than female lambs.

### MSTN variation in the studied breeds

PCR amplification produced a 386 bp PCR product from the first intron of the *MSTN* gene in different sheep breeds, including Barki (accession no. MT361503), Ossimi (accession no. MT361504), Rahmani (accession No. MT361505), and Najdi (accession no. MT361506). These sequences were analyzed to detect SNPs in the studied sheep breeds (Figure-1A and B). FourSNPs were detected: c.18 G>T, c.241 T>C, c.243 G>A, and c.259 G>T. The most interesting SNPs detected were c.159 A>T and c.173 T>G, which showed one genotype (100% AA and 100% TT, respectively) (monomorphism) in Ossimi, Rahmani, and Najdi sheep and different genotypes (polymorphism) in Barki sheep (Table-2). Table-3 presents the genotypic and allelic frequencies of *MSTN* variants in the studied sheep breeds.

**Figure-1A:**
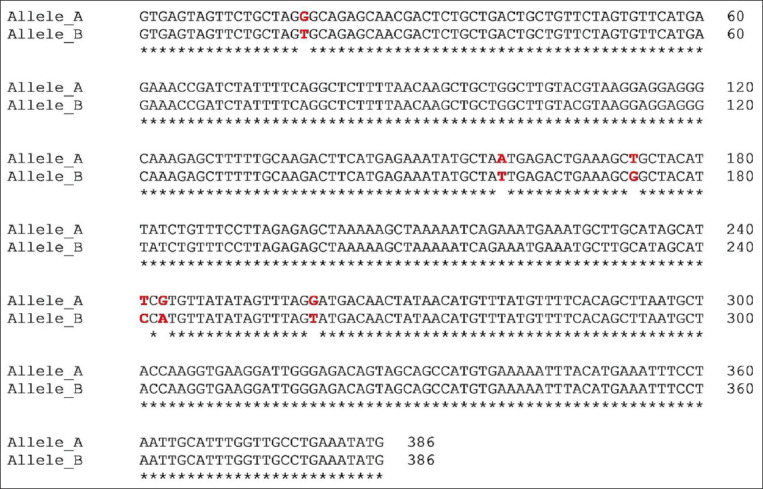
Polymerase chain reaction product sequences of two different alleles in Egyptian Braki sheep, showing single-nucleotide polymorphisms positions in red color.

**Figure-1B:**
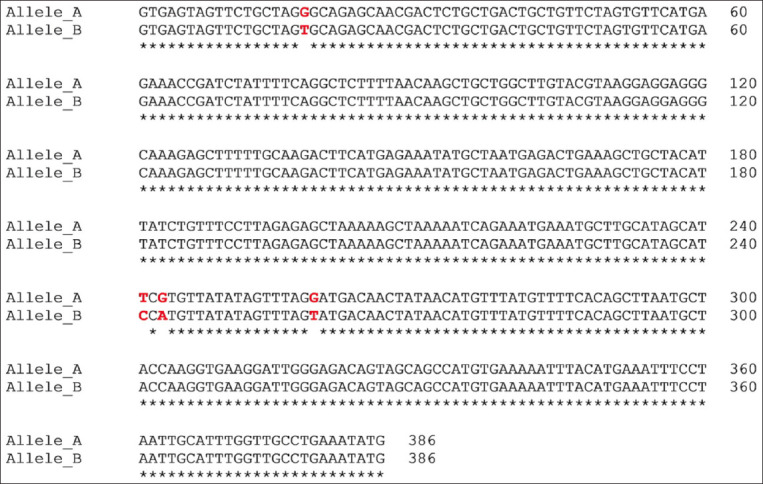
Polymerase chain reaction product sequences of two different alleles in Egyptian (Ossimi and Rahmani) and Saudi Arabia (Najdi) sheep breeds, showing single-nucleotide polymorphisms positions in red color.

**Table-2:** SNPs positions and genotype frequencies detected in *MSTN* intron 1 of different sheep breeds.

SNP position	SNPs	Barki (%)	Ossimi (%)	Rahmani (%)	Najdi (%)	Chromatogram
c. 18 G>T	GG	39	33	44	31	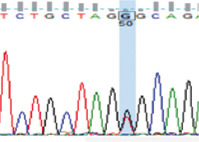
	GT	47	49	45	49	
	TT	14	18	11	20	
c. 159 A>T	AA	69	100	100	100	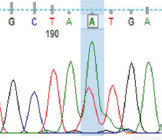
	AT	28	0	0	0	
	TT	3	0	0	0	
c. 173 T>G	TT	69	100	100	100	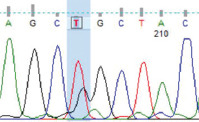
	TG	28	0	0	0	
	GG	3	0	0	0	
c. 241 T>C	TT	39	64	56	20	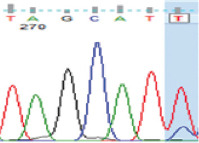
	TC	47	32	38	49	
	CC	14	4	6	31	
c. 243 G>A	GG	39	64	56	20	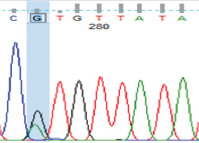
	GA	47	32	38	49	
	AA	14	4	6	31	
c. 259G>T	GG	39	33	44	20	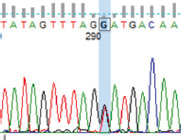
	GT	47	49	45	49	
	TT	14	18	11	31	

SNPs=Single nucleotide polymorphisms, *MSTN*=*Myostatin*

**Table-3:** Genotypic and allelic frequencies of *Myostatin* variants in the studied sheep breeds.

SNP	Allelic frequency %		Genotypic frequency %		
SNPc. 18 G>T	G	T	GG	GT	TT
Barki	63	37	39	47	14
Ossimi and Rahmani	63	37	39	47	14
SNPc. 159 A>T	A	T	AA	AT	TT
Barki	83	17	69	28	3
Ossimi and Rahmani	100	0	100	0	0
SNPc. 173 T>G	T	G	TT	TG	GG
Barki	83	17	69	28	3
Ossimi and Rahmani	100	0	100	0	0
SNPc. 241 T>C	T	C	TT	TC	CC
Barki	63	37	39	47	14
Ossimi and Rahmani	76	24	55	36	9
SNPc. 243 G>A	G	A	GG	GA	AA
Barki	63	37	39	47	14
Ossimi and Rahmani	76	24	55	36	9
SNPc. 259 G>T	G	T	GG	GT	TT
Barki	63	37	39	47	14
Ossimi and Rahmani	63	37	39	47	14

### Effect of MSTN genotype on the studied traits

Among the six identified SNPs across the amplified region of *MTSN* in the three Egyptian sheep breeds, two SNPs tended to significantly influence one of the studied traits. The SNP c.18 G>T (*rs119102825*, also known as c.373+18G>T) and SNP c.241 T>C (*rs119102826*, also known as c.373+241T>C) showed significant associations with BW (p=0.05) and ADG (p=0.03), respectively. Lambs that carried the GG genotype at position 18 tended to have higher BW (3.55 kg) compared with other genotypes, whereas the TT carriers at position 241 tended to have higher ADG (101 g/day) compared with other genotypes. The rest of the SNPs did not show any significant association with the studied traits (Table-4).

**Table-4:** Effect of the *Myostatin* gene genotypes on growth traits in Egyptian sheep.

Genotype	Trait (LS means±standard error)		

	BW	FW	ADG
SNP c. 18 G>T			
GG	3.55±0.04	51.56±1.31	100.96±2.26
GT	3.12±0.05	47.87±0.86	101.67±2.73
TT	3.22±0.08	49.07±0.71	98.49±4.18
Significance	0.05*	0.06	0.62
SNP c. 241 T>C			
CC	3.32±0.05	49.07±0.83	85.86±4.12
CT	3.22±0.06	47.87±1.00	97.62±4.83
TT	3.45±0.09	51.56±1.53	101.13±7.61
Significance	0.11	0.13	0.03*
SNPc. 243 G>A			
GG	3.23±0.03	42.20±0.6	97.90±1.85
AG	3.31±0.06	48.14±1.06	103.84±3.18
AA	3.32±0.07	49.00±1.20	102.88±3.86
Significance	0.72	0.8	0.18
SNP c. 259 G>T			
GG	3.25±0.03	49.00±0.60	97.91±1.80
GT	3.31±0.06	48.41±1.04	103.84±3.18
TT	3.32±0.07	49.20±1.20	102.82±3.86
Significance	0.72	0.81	0.18
SNP c. 159 A>T			
GG	3.24±0.21	48.94±0.80	99.90±2.53
GT	3.54±0.21	51.68±3.56	100.51±11.17
TT	3.31±0.04	48.98±0.81	100.51±11.17
Significance	0.51	0.57	0.96
SNP c. 173 T>G			
GG	3.27±0.33	46.54±5.42	99.91±3.86
GT	3.54±0.133	51.68±5.36	100.51±17.00
TT	3.30±0.07	48.98±1.23	100.51±17.00
Significance	0.75	0.78	0.96

^1^BW=Birth weight (kg), FW=Full weight (kg), ADG=Average daily gain (g/day). *Significance level (p<0.05)

## Discussion

*MSTN* encodes a negative growth factor that inhibits both the terminal differentiation of myoblasts and the proliferation of myogenic cells [31,32]. It was reported as a candidate gene for improved muscle growth in livestock [33], which is positively correlated with the growth performance of the animal. Two mutations in the gene were identified with high frequency as muscular hypertrophy alleles in Belgian Blue and Piedmontese cattle. Polymorphisms were also reported in this gene in different sheep breeds worldwide [9,21-23,34,35]. Subsequently, these reports attempted to correlate these variants with important growth and carcass traits of such breeds. Similarly, Shafey *et al*. [36], Othman *et al*. [37] reported genetic polymorphisms in the *MSTN* gene in Egyptian sheep. To the best of our knowledge, our results show the first association between these polymorphisms and growth performance in Egyptian sheep breeds. Moreover, we report novel and breed-specific variants in the Egyptian major sheep breeds.

Growth performance is a complex trait that is likely to be regulated by multiple genes. Therefore, it is always of primary concern in breeding schemes to determine an animal’s breeding value [38]. In general, identifying genetic markers for growth traits are an initial and crucial step to establish a marker-assisted selection system [39]. The main determinants of fast growth in mammals are increased muscle cell growth and proliferation. In general, the estimates of growth traits included in the present study were consistent with those reviewed by Elshennawy [40] for the same breeds. However, slightly higher estimates were reported in Barki sheep by Sallam [41] (3.33 kg and 140 g/day compared with 2.93 kg and 90.125 g/day for BW and ADG, respectively, in the present study).

Despite the tendency of the sex of the animal to influence growth traits in sheep [37,42], these effects were not significant in our results for BW. This may be due to the limited sample size population used in the present study. In agreement, the sex of the animal had no significant effect on the growth traits in other sheep breeds such as the Moghani breed [43]. Conversely, other studies have reported a significant effect of sex on growth traits [37,44]. However, phenotypic variations were observed between breeds in BW, as Rahmani and Ossimi sheep had higher BW than did Barki sheep. These breed and sex differences in FW were significant, in agreement with those reported by Othman *et al*. [37].

The previous studies [22,45] showed that the exons and 3′-untranslated regions of *MSTN* were monomorphic in the studied sheep breeds. Conversely, our screening of the first exon of the *MSTN* gene showed polymorphism in the studied breeds, which was in agreement with Gan [21], Clop *et al*. [46], who reported that the first intron of the ovine *MSTN* gene was highly polymorphic in different sheep breeds. Hickford *et al*. [35] detected polymorphisms in the first intron of the *MSTN* gene and reported associations between these alleles and carcass traits in New Zealand Romney sheep. Similarly, Sjakste *et al*. [23] identified several SNPs in the same fragment of the *MTSN* gene in Latvian Dark head sheep, suggesting that polymorphisms in this non-coding region can affect regulatory elements.

In this study, genetic diversity analysis revealed that four mutual polymorphic sites were detected in four different sheep breeds (Barki, Ossimi, Rahmani, and Najdi) at nucleotide positions G18, T241, G243, and G259 after sequencing the amplified fragments. Consistently, polymorphisms in the first intron of the *MSTN* gene were identified in the Iranian Makuei sheep breed [47] and in the Kamieniec and Pomeranian sheep breeds [28]. By contrast, Soufy *et al*. [48] reported that the first intron was monomorphic, and all samples showed the same genotypes in Sanjabi sheep; similarly, Nada *et al*. [49] reported that all samples of the Egyptian (Barki, Ossimi, and Rahmani) and Saudi (Najdi and Harri) breeds showed the same genotype for exon 3 of the *MTSN* gene. However, Sahu [50] reported the first variations in exon 3 of the *MSTN* gene in Nilagiri sheep in South Africa, such as g.5622 G>C. Interestingly, the c.159 A>T mutation showed polymorphism between an A and a T allele in Barki sheep, whereas it was monomorphic (AA) in Ossimi, Rahmani, and Najdi breeds. Similarly, polymorphism at c.173 T>G showed three different genotypes (TT, TG, and GG) in Barki and one genotype (TT) in the other breeds examined. This may explain the higher heterozygosity in this breed. Similarly, higher heterozygosity has been reported in the Barki breed than in the Ossimi and Rahmani breeds [36]. Higher genetic diversity observed in the present study may be due to the intensive crossing processes in Barki sheep in comparison with other breeds. Moreover, this higher variability in the genetics of Barki has made this breed to be more adapted to the harsh conditions of the Egyptian desert, which is the predominant region in which this breed is cultivated [51]. Increasing the sample size may find additional polymorphisms in subsequent analyses [41].

Rather than relying on traditional breeding approaches to improve the growth traits in sheep, adopting genetic markers are an efficient adjunct tool to successfully achieve this improvement [20]. Reportedly, variants in the non-coding regions of the genome can influence phenotypes by affecting gene regulation [22,23]; for example, G/T transversion at c.373+18 could functionally affect transcript splicing. Polymorphisms in the non-coding region of *MSTN* were reported to affect growth and carcass traits [21,34,35] in different sheep breeds worldwide. Consistently, the two SNPs (*rs119102825* and *rs119102826*) identified in this study were previously reported as significantly associated variants with several growth traits in several sheep breeds both in New Zealand sheep [9] and in Polish Merino sheep [22]. Accordingly, our results suggest that polymorphisms within *MSTN* significantly influence growth traits in the Egyptian sheep breeds.

## Conclusion

In the present study, we shed light on the *MSTN* gene as a potential promising genetic marker to improve growth traits in the major sheep breeds in Egypt. Sequence analysis of the first intron of the *MSTN* gene identified six SNPs in the studied breeds. Four mutual SNPs were identified: c.18 G>T, c.241 T>C, c.243 G>A, and c.259 G>T, as well as two SNPs c.159 A>T and c.173 T>G that were monomorphic (AA and TT, respectively) in the Ossimi, Rahmani, and Najdi breeds and polymorphic in the Barki breed. Association analysis revealed that c.18 G>T and c.241 C>T significantly associated (p<0.05) with BW and ADG, respectively. Our results suggest that polymorphisms within *MSTN* significantly influence growth traits in the Egyptian sheep breeds. We strongly ­recommend reanalyzing *MSTN* variants using larger sample sizes to detect these polymorphisms and increasing the power of the current investigation.

## Authors’ Contributions

KFM conceived the idea and designed the experiment. NMO performed DNA sequence and variants analysis. AMS performed statistical analysis. HIS and MAA performed the experiments. KFM, NMO, HIS, and AMS wrote the manuscript. All the authors revised, read, and approved the final manuscript.

## References

[1] El Fiky Z.A, Hassan G.M, Nassar M.I (2017). Genetic polymorphism of growth differentiation factor 9 (GDF9) gene related to fecundity in two Egyptian sheep breeds. J. Assist. Reprod. Genet.

[2] Galal S, Abdel-Rasoul F, Anous M.R, Shaat I, Iniguez L.C (2005). On station characterization of small ruminant breeds in Egypt. Characterization of Small Ruminant Breeds in West Asia and North Africa.

[3] Abdel-Moneim A.Y (2009). Body and carcass characteristics of Ossimi, Barki and Rahmani ram lambs raised under intensive production system. Egypt. J. Sheep Goat Sci.

[4] Aljumaah R.S, Al-Shaikh M.A, Kibogo H, Kwallah A, Jianlin H, Hanotte O, Musthafa M.M, Marikar F.M.M (2014). Genetic relationships among four Saudi Arabian sheep populations. Iran. J. Appl. Anim. Sci.

[5] Amare T, Goshu G, Tamir B (2018). Flock composition, breeding strategies and farmers'traits of interest evaluation of Wollo highland sheep and their F 1 crosses. J. Anim. Sci. Technol.

[6] Crispo M, Mulet A.P, Tesson L, Barrera N, Cuadro F, dos Santos-Neto P.C, Nguyen T.H, Creneguy A, Brusselle L, Anegon I, Menchaca A (2015). Efficient generation of myostatin knock-out sheep using CRISPR/Cas9 technology and microinjection into zygotes. PLoS One.

[7] McPherron A.C, Lawler A.M, Lee S.J (1997). Regulation of skeletal muscle mass in mice by a new TGF-beta superfamily member. Nature.

[8] Hadjipavlou G, Matika O, Clop A, Bishop S.C (2008). Two single nucleotide polymorphisms in the myostatin (GDF8) gene have significant association with muscle depth of commercial *Charollais* sheep. Anim. Genet.

[9] Han J, Forrest R.H, Hickford J.G (2013). Genetic variations in the myostatin gene (MSTN) in New Zealand sheep breeds. Mol. Biol. Rep.

[10] Casas E, Kehrli M.E (2016). A review of selected genes with known effects on performance and health of cattle. Front. Vet. Sci.

[11] Zhang X.X, Ran J.S, Lian T, Li Z.Q, Yang C.W, Jiang X.S, Du H.R, Cui Z.F, Liu Y.P (2019). The single nucleotide polymorphisms of myostatin gene and their associations with growth and carcass traits in Daheng broiler. Braz. J. Poult. Sci.

[12] Dall'Olio S, Fontanesi L, Nanni Costa L, Tassinari M, Minieri L, Falaschini A (2010). Analysis of horse myostatin gene and identification of single nucleotide polymorphisms in breeds of different morphological types. J. Biomed. Biotechnol.

[13] Guo R, Wan Y, Xu D, Cui L, Deng M, Zhang G, Jia R, Zhou W, Wang Z, Deng K, Huang M, Wang F, Zhang Y (2016). Generation and evaluation of Myostatin knock-out rabbits and goats using CRISPR/Cas9 system. Sci. Rep.

[14] Amthor H, Macharia R, Navarrete R, Schuelke M, Brown S.C, Otto A, Voit T, Muntoni F, Vrbova G, Partridge T, Zammit P, Bunger L, Patel K (2007). Lack of myostatin results in excessive muscle growth but impaired force generation. Proc. Natl. Acad. Sci. U. S. A.

[15] Deng B, Zhang F, Wen J, Ye S, Wang L, Yang Y, Gong P, Jiang S (2017). The function of myostatin in the regulation of fat mass in mammals. Nutr. Metab.

[16] Grade C.V.C, Mantovani C.S, Alvares L.E (2019). Myostatin gene promoter:Structure, conservation and importance as a target for muscle modulation. J. Anim. Sci. Biotechnol.

[17] Fan B, Du Z.Q, Gorbach D.M, Rothschild M.F (2010). Development and application of high-density SNP arrays in genomic studies of domestic animals. Asian Aust. J. Anim. Sci.

[18] Kwon J.M, Goate A.M (2000). The candidate gene approach. Alcohol. Res. Health.

[19] Sallam A.M, Zare Y, Shook G, Collins M, Kirkpatrick B.W (2018). A positional candidate gene association analysis of susceptibility to paratuberculosis on bovine chromosome 7. Infect. Genet. Evol.

[20] Sallam A.M, Gad-Allah A.A, Al-Bitar E.M (2020). Association analysis of the ovine KAP6-1 gene and wool traits in Barki sheep. Anim. Biotechnol.

[21] Gan S.Q, Du Z, Liu S.R, Yang Y.L, Shen M, Wang X.H, Yin J.L, Hu X.X, Fei J, Fan J.J, Wang J.H, He Q.H, Zhang Y.S, Li N (2008). Association of SNP haplotypes at the myostatin gene with muscular hypertrophy in sheep. Asian Aust. J. Anim. Sci.

[22] Grochowska E, Borys B, Mroczkowski S (2019). Effects of intronic SNPs in the myostatin gene on growth and carcass traits in colored Polish merino sheep. Genes (Basel).

[23] Sjakste T, Paramonova N, Grislis Z, Trapina I, Kairisa D (2011). Analysis of the single-nucleotide polymorphism in the 5'UTR and part of intron I of the sheep MSTN gene. DNA Cell. Biol.

[24] Boman I.A, Klemetsdal G, Nafstad O, Blichfeldt T, Vage D.I (2010). Impact of two myostatin (MSTN) mutations on weight gain and lamb carcass classification in Norwegian White Sheep (*Ovis aries*). Genet. Sel. Evol.

[25] Yamada A.K, Verlengia R, Bueno Junior C.R (2012). Myostatin:Genetic variants, therapy and gene doping. Braz. J. Pharm. Sci.

[26] Zhang C, McFarlane C, Lokireddy S, Masuda S, Ge X, Gluckman P.D, Sharma M, Kambadur R (2012). Inhibition of myostatin protects against diet-induced obesity by enhancing fatty acid oxidation and promoting a brown adipose phenotype in mice. Diabetologia.

[27] Kolenda M, Grochowska E, Milewski S, Mroczkowski S (2019). The association between the polymorphism in the myostatin gene and growth traits in Kamieniec and Pomeranian sheep breeds. Small Ruminant Res.

[28] Miller S.A, Dykes D.D, Polesky H.F (1988). A simple salting out procedure for extracting DNA from human nucleated cells. Nucleic Acids Res.

[29] Altschul S.F, Gish W, Miller W, Myers E.W, Lipman D.J (1990). Basic local alignment search tool. J. Mol. Biol.

[30] Thompson J.D, Higgins D.G, Gibson T.J (1994). CLUSTAL W:Improving the sensitivity of progressive multiple sequence alignment through sequence weighting, position-specific gap penalties and weight matrix choice. Nucleic Acids Res.

[31] Thomas M, Langley B, Berry C, Sharma M, Kirk S, Bass J, Kambadur R (2000). Myostatin, a negative regulator of muscle growth, functions by inhibiting myoblast proliferation. J. Biol. Chem.

[32] Wiener P, Woolliams J.A, Frank-Lawale A, Ryan M, Richardson R.I, Nute G.R, Wood J.D, Homer D, Williams J.L (2009). The effects of a mutation in the myostatin gene on meat and carcass quality. Meat. Sci.

[33] Kambadur R, Sharma M, Smith T.P, Bass J.J (1997). Mutations in myostatin (GDF8) in double-muscled Belgian blue and Piedmontese cattle. Genome Res.

[34] Kijas J.W, McCulloch R, Edwards J.E, Oddy V.H, Lee S.H, van der Werf J (2007). Evidence for multiple alleles effecting muscling and fatness at the ovine GDF8 locus. BMC Genet.

[35] Hickford J.G, Forrest R.H, Zhou H, Fang Q, Han J, Frampton C.M, Horrell A.L (2010). Polymorphisms in the ovine myostatin gene (MSTN) and their association with growth and carcass traits in New Zealand Romney sheep. Anim. Genet.

[36] Shafey H.I, Mahrous K.F, Hassanane M.S, Mordy M.A, Rushdi H.E (2014). Genetic polymorphism of myostatin and insulin-like growth factor binding protein-3 genes in Egyptian sheep breeds. Global Vet.

[37] Othman O.E, Balabel E.A, Mahfouz E.R (2016). Genetic characterization of myostatin and callipyge genes in Egyptian small ruminant breeds. Biotechnology.

[38] Malik Z.S, Dalal D.S, Dahiya S.P, Patil C.S, Dahiya R (2016). Genetic analysis of growth traits in Harnali sheep. Vet. World.

[39] Ekegbu U.J, Burrows L, Amirpour-Najafabadi H, Zhou H, Hickford J.G.H (2019). Gene polymorphisms in PROP1 associated with growth traits in sheep. Gene.

[40] Elshennawy M, Gabina D (1995). Sheep development program in Egypt. Strategies for Sheep and Goat Breeding.

[41] Sallam A.M, Ibrahim A.H, Alsheikh S.M (2019). Genetic evaluation of growth in Barki sheep using random regression models. Trop. Anim. Health Prod.

[42] Ibrahim A.H.M (2019). Association of growth performance and body conformational traits with BMP4 gene variation in Barki lambs. Growth Factors.

[43] Lavvaf A, Noshary A, Keshtkaran A (2007). Environmental and genetic effects on early growth traits in Moghani sheep breeds. Pak. J. Biol. Sci.

[44] Esenbuga N, Dayıoglu H (2002). Effects of some environmental factors on growth traits of Awassi and Redkaraman lambs. Turk. J. Vet. Anim. Sci.

[45] Dehnavi E, Ahani Azari M, Hasani S, Nassiry M.R, Mohajer M, Khan Ahmadi A, Shahmohamadi L, Yousefi S (2012). Polymorphism of myostatin gene in intron 1 and 2 and exon 3, and their associations with yearling weight, using PCR-RFLP and PCR-SSCP techniques in zel sheep. Biotechnol. Res. Int.

[46] Clop A, Marcq F, Takeda H, Pirottin D, Tordoir X, Bibe B, Bouix J, Caiment F, Elsen J.M, Eychenne F, Larzul C, Laville E, Meish F, Milenkovic D, Tobin J, Charlier C, Georges M (2006). A mutation creating a potential illegitimate microRNA target site in the myostatin gene affects muscularity in sheep. Nat. Genet.

[47] Farhadian M, Hashemi A (2016). Molecular characterization and phylogeny based analysis of intron i sequence of myostatin (MSTN) gene in Iranian Makuei sheep breed. Ann. Anim. Sci.

[48] Soufy B, Mohammadabadi M.R, Shojaeyan K, Baghizadeh A, Ferasaty S, Askari N, Dayani O (2009). Evaluation of myostatin gene polymorphism in Sanjabi sheep by PCR-RFLP method. Anim. Sci. Res.

[49] Nada E, Mahrous K.F, Salem L.M (2013). Genetic polymorphism detection in four genes in Egyptian and Saudi sheep breeds. World Appl. Sci. J.

[50] Sahu A.R (2019). Novel report on mutation in exon 3 of myostatin (MSTN) gene in Nilagiri sheep:An endangered breed of South India. Trop. Anim. Health Prod.

[51] Abousoliman I, Reyer H, Oster M, Murani E, Mourad M, Rashed M.A, Mohamed I, Wimmers K (2020). Analysis of candidate genes for growth and milk performance traits in the Egyptian Barki sheep. Animals (Basel).

